# Immobilisation and Release of Radical Scavengers on Nanoclays for Chemical Reinforcement of Proton Exchange Membranes

**DOI:** 10.3390/membranes10090208

**Published:** 2020-08-28

**Authors:** Alia Akrout, Aude Delrue, Marta Zatoń, Fanny Duquet, Francesco Spanu, Mélanie Taillades-Jacquin, Sara Cavaliere, Deborah Jones, Jacques Rozière

**Affiliations:** 1 Institute Charles Gerhardt Montpellier, UMR CNRS 5253, Aggregates Interfaces and Materials for Energy, University of Montpellier, CEDEX 5, 34095 Montpellier, France; alia.akrout@etu.umontpellier.fr (A.A.); delrue.a@live.fr (A.D.); marta.zaton@umontpellier.fr (M.Z.); fanny.duquet@umontpellier.fr (F.D.); francesco.spanu@umontpellier.fr (F.S.); melanie.taillades-jacquin@umontpellier.fr (M.T.-J.); deborah.jones@umontpellier.fr (D.J.); jacques.roziere@umontpellier.fr (J.R.); 2 Institut Universitaire de France (IUF), CEDEX 05, 75231 Paris, France

**Keywords:** proton exchange membrane fuel cells, radical scavengers, halloysite, cerium oxide

## Abstract

Mechanical and chemical stability of proton exchange membranes are crucial requirements for the development of fuel cells for durable energy conversion. To tackle this challenge, bi-functional nanoclays grafted with amino groups and with embedded radical scavengers, that is, CeO_2_ nanoparticles were incorporated into Aquivion^®^ ionomer. The composite membranes presented high proton conductivity and increased stability to radical attack compared to non-modified Aquivion membranes, demonstrating the effectiveness of the approach based on radical scavenger immobilisation and release from clay nanocontainers.

## 1. Introduction

Despite the significant progress in proton exchange membrane development for fuel cells over the past decade [1,2,3], improvement of their durability to meet transport application targets [4] is still a great challenge. Advances have also been made in understanding the origins of perfluorosulfonic acid (PFSA) membrane degradation, leading to the development of novel strategies and materials for its mitigation [5,6,7,8,9].

Mechanical degradation of a proton exchange membrane during fuel cell operation occurs as a consequence of dimensional changes due to hydration/dehydration or to the variation in stack compression, leading to crack propagation and pinhole formation [7,10,11]. Mechanical stability has become a greater challenge with the use of thinner membranes to benefit from the advantages of low membrane resistance and improved water transport. Being related to mechanical and chemical degradation of the membrane, failure stress and tear resistance are used as indicators of the durability of membrane-electrode assemblies [12]. Several chemical and physical routes have been employed to increase membrane mechanical resistance, including thermal annealing [13,14] and chemical cross-linking [15] or preparation of composite membranes incorporating polymer reinforcements [16,17], electrospun nanofibres [18,19,20], carbon nanotubes [21,22], inorganic particles [23,24,25,26] and clays [27,28,29,30].

Chemical degradation of PFSA membranes is induced by the attack of free radicals (HO•, HOO•) generated in the fuel cell environment [31]. Hydroxyl and hydroperoxyl radicals are the products of decomposition of hydrogen peroxide formed at the cathode [32]. In the presence of traces of iron or other multivalent metal ions (e.g., Cu^2+^ or Ti^3+^) originated from corrosion of the cell, stack materials or humidifiers, the reaction of H_2_O_2_ decomposition is catalysed to produce radicals by the Fenton reaction [33,34]. The formed HO• and HOO• attack specific sites on the polymer side or main chains, leading to membrane thinning and pinhole formation due to defragmentation via the unzipping mechanism and fluoride ion release [3,8] and decrease in the ion exchange capacity and consequently in the proton conductivity [6,35]. The former and in particular the fluoride emission rate (FER), that is, the release of fluoride ions per unit area with time from a membrane upon an accelerated stress test (AST) for example, Fenton’s reaction, is the conventional indicator of the extent of membrane degradation [36]. To mitigate chemical degradation of membranes the incorporation of radical scavengers, organic (terephthalic acid, λ-tocopherol) as well as inorganic (metal oxides) has been demonstrated to be an effective approach [37,38,39,40]. In particular, cerium ions or oxide nanoparticles have been thoroughly investigated due to their faster reaction rate they have with free radicals than that of the free radicals with the polymer membrane [37] and their fast reversible redox reaction in aqueous conditions [41].

Despite the effective stabilisation of the membranes as indicated by strong decrease of the FER upon accelerated stress tests [42,43], the issue of their stability in acidic environment arose [31]. Ce ions can migrate in the operating fuel cell, favoured by concentration gradients and water flow and are leached into exhaust water [44,45,46] The possibility of immobilising them in the membrane while allowing their controlled release would be a valuable approach to overcome this limitation and make the scavenging effect more durable [31,47,48,49].

As already mentioned, natural, synthetic and modified clays have been thoroughly used for the preparation of composite membranes, in particular to improve their dimensional and mechanical properties and to provide physical barriers to gas crossover [50,51]. Furthermore, clay materials can be used to immobilise radical scavengers to avoid their elution, playing the role of mechanical as well as chemical stabilisers. Tubular nanoclays and in particular halloysite nanotubes (HNTs) have already been used as containers for the encapsulation and the sustained release of multiple entities (molecules, particles…) especially in the biomedical field [52,53,54]. HNTs are a naturally occurring aluminosilicate (Al_2_Si_2_O_5_(OH)_4_^.^nH_2_O) belonging to the kaolinite group with nanometric dimensions and a hollow tubular structure with an inner *lumen*. The chemical compositions of the inner and outer surfaces of HNTs are different, being formed by Al-OH and Si-O sheets, respectively, a situation that allows regioselective functionalisation [55]. Moreover, halloysite nanotubes have a significant mechanical and thermal reinforcing effect on polymer matrices [56], which makes them promising components of proton exchange membranes. 

In this work, HNTs were used as nanocontainers to encapsulate and release cerium oxide nanoparticle radical scavengers to prevent chemical degradation of a PFSA membrane by radical attack. We report the preparation and characterisation of a composite proton exchange membrane based on Aquivion^®^ and the cerium oxide-functionalised HNTs (CeO_2_@HNT). Furthermore, amino moieties were grafted on the outer surface of the HNTs using an aminosilane coupling agent in order to improve their compatibility with the acidic ionomer and ensure high dispersion and homogeneity. The bi-functional nanoclays, labelled CeO_2_@HNT-NH_2_, were characterised by X-ray diffraction (XRD), X-ray fluorescence (XRF), infrared spectroscopy (IR), thermogravimetric analysis (TGA), zeta potential, solid-state ^29^Si MAS NMR (magic-angle spinning nuclear magnetic resonance) and electron microscopies to evaluate the composition and degree of the inner/outer functionalisation. The composite membranes incorporating such HNTs were characterised for their in-plane proton conductivity and mechanical properties upon strain/stress. They were also submitted to the Fenton reaction to assess the effect of the functionalised clays embedding cerium oxide on the chemical stability, as monitored by the FER. The HNT loading in PFSA was adjusted to find a compromise between chemical stability and proton conductivity adapted for their application in proton exchange membrane fuel cells (PEMFC).

## 2. Materials and Methods

### 2.1. Materials

All the following chemicals were purchased from Sigma Aldrich (St Louis, USA) and used as received—halloysite clay nanotubes (HNTs) mined from the High Purity Dragon Mine in Silver City, Utah (USA), oxalic acid solution (1 mol/L), cerium (III) nitrate hexahydrate (99%), anhydrous ethanol, (3-aminopropyl)trimethoxysilane (97%), anhydrous toluene, N,N-dimethylacetamide solution (anhydrous, 99.8%), 2-propanol, hydrogen peroxide (30% volume), ammonium iron(II) sulfate ((NH_4_)_2_Fe(SO_4_)_2_(H_2_O)_6_) (99.997%), sulfuric acid (97%), total ionic strength adjustment buffer III solution (TISAB III). Composite membranes were prepared using Aquivion^®^ ionomer (830 EW 24 wt% suspension in water) purchased from Solvay (Brussels, Belgium). 

### 2.2. Preparation of CeO_2_ Embedded and Amino Functionalised HNT and Their Characterisation

#### 2.2.1. HNT Pre-Treatment

Pre-treatment with acid was applied as follows to leach the iron ions naturally present in HNTs—1 g of HNTs was dispersed in a 10 mL solution of 0.45 M oxalic acid and the dispersion was stirred for 3 hs at 80 °C and then filtered using a 0.22 µm polyvinylidene fluoride (PVDF) filter (Durapore membrane filters). The obtained HNTs were rinsed three times with MilliQ grade water and dispersed in 10 mL of ultra-pure water by ultrasonication (Branson digital sonication) at 5 W for 10 min. The obtained HNTs were dried for 16 h at 80 °C and then characterised by XRF to quantify the amount of the eventual residual iron. 

#### 2.2.2. Preparation of CeO_2_@HNT

A 3 M solution of Ce (NO_3_)_3_ 6H_2_O was prepared in EtOH. The treated HNTs were submitted to vacuum-cycling of 0.12 g of HNTs dispersed in 40 mL of this solution. The suspension was kept under vacuum for 1 h in order to remove the air present in the *lumen* and to facilitate insertion of cerium nitrate solution. The suspension was then cycled back to atmospheric pressure. This process was repeated three times. The HNTs embedding cerium nitrate, labelled Ce@HNTs, were separated from the solution by filtration on a PVDF filter (0.22 µm) and rinsed twice with methanol (anhydrous, 99.8%) to ensure the removal of any cerium nitrate that might be present on their surface. Finally, the Ce@HNTs powder was dried at 80 °C for 16 hs. In order to ensure the stability of embedded cerium and prevent its leaching, Ce@HNTs were heat treated in air at 300 °C for 5 hs to convert the cerium nitrate to cerium oxide nanoparticles [57], embedded within the clay *lumen*, leading to the material labelled CeO_2_@HNTs.

#### 2.2.3. Surface Functionalisation of CeO_2_@HNT

Organosilane modified CeO_2_@HNTs were prepared by adding 0.3 g of CeO_2_@HNTs to a solution of 1.5 mL of APTMS in 12.5 mL of dry toluene. The suspension obtained was dispersed using an ultrasonic bath during 30 min and then transferred under reflux at 120 °C for 20 hs. A calcium chloride drying tube was used to ensure a dry environment. The suspension was then filtered and washed with toluene six times to afford CeO_2_@HNT-NH_2_ that was dried at 120 °C for 16 hs. 

#### 2.2.4. Physico-Chemical Characterisation of HNT

The morphology of halloysite nanotubes (HNT) was investigated by field emission-scanning electron microscopy (FE-SEM) using a Hitachi S-4800 microscope (Hitachi Europe SAS, Velizy, France). Data analysis and particle size distribution of HNT were performed using an image processing software Image J 1.48 v (U. S. National Institutes of Health, Bethesda, MD, USA). Halloysite nanotubes were analysed by transmission electron microscopy (TEM) using a JEOL 2200FS (Source: FEG) microscope operating at 200 kV equipped with a CCD camera Gatan USC (16 MP) (JEOL, Tokyo, Japan).

The different components present were identified by X-ray diffraction (XRD, PANalytical X’Pert in Bragg-Brentano configuration with CuK_α_ radiation, (Malvern Panalytical, Cambridge, United Kingdom from 10 to 70° with a step angle of 0.032°.

Pristine and functionalised HNTs were characterised by thermogravimetric analysis (TGA, Netzsch, Selb, Germany) with a NETZSCH STA 409 PC from 20 °C to 1200 °C with a ramp of 10 °C min^−1^ under nitrogen.

Wavelength dispersive X-Ray Fluorescence (XRF) spectrometer (Axios max, PANalytical, Cambridge, United Kingdom) was used for quantitative elemental analysis of materials. The X-ray tube in the spectrometer had an Rh anode and operated at a maximum power of 4 kW with a maximum voltage of 60 kV or maximum current of 160 mA. To obtain high resolution fluorescence spectra, eight LiF200 dispersive crystals were used. The measurements were performed under vacuum in fourteen different scans. Each scan covered a range of the expected elements and the peak areas were determined using Spectra Evaluation of SuperQ software. Samples were prepared by grinding 400 mg of sample and pressing it to obtain a pellet of 32 mm diameter. The same protocol was used to prepare the standards to obtain a calibration line. For iron determination, standards were prepared using a mixture containing alumina (prepared in the laboratory) and varying amounts of iron oxide (Sigma Aldrich, Saint Louis, MO, USA, nanopowder, <50 nm particle size—0.1, 0.2, 0.3 and 0.5 wt%). For cerium determination, standards were prepared with halloysite and varying amounts of CeO_2_ powder (Sigma Aldrich, Saint Louis, MO, USA, <5 µm, 99.9%: 1, 3, 5, 7 and 10 wt%).

Surface charge of the clays was determined using a Malvern Zetasizer 3000HSa (Malvern Panalytical, Cambridge, United Kingdom) at pH 7 after preparing a dispersion of 1 mg of HNTs in 5 mL of deionised water. 

Infrared spectroscopy was used to determine the surface functionalities of the different halloysites upon acidic treatment and functionalisation using a spectrum two spectrometer (Perkin Elmer, Waltham, MA, USA). 5 mg of the samples were analysed in powder form.

The solid-state ^29^Si NMR spectra of APTMS functionalised clays were recorded on a 300 MHz VARIAN VNMRS300 300 MHz spectrometer (7.05 Tesla “Wide Bore” magnet, LabX, Midland, ON, Canada). A VARIAN T^3^ MAS (Magic Angle Spinning, LabX, Midland, ON, Canada) probe with 7.5 mm ZrO_2_ rotors was used. The measurements were carried out with the CPMAS technique (non-quantitative) and Single Pulse (quantitative/single pulse ^29^Si followed by ^1^H decoupling). For CPMAS, a π/2 pulse of 6 µS, a contact time of 3 ms and a recycling time of 3 s were used. For the Single Pulse and to guarantee the quantitative analysis a 2 µs π /6 pulse and a recycling time of 60 s were used. The samples rotated at 5 kHz. The chemical shift values were calibrated using Q8M8H (octakis (dimethylsiloxy) octasilsesquioxane) as a secondary reference (line at −2.25 ppm). The acquisition window was 50 kHz and the filtering (line broadening) 50H. The assignment of the chemical shifts and the identification of the materials were performed based on a model spectrum obtained with the ChemBio Draw software (Ultra 14.0, PerkinElmer, Waltham, MA, USA).

### 2.3. Membrane Preparation and Characterisation

Composite membranes were prepared by casting Aquivion^®^ 830 EW (10 wt%) dispersion containing different amounts of CeO_2_@HNT-NH_2_ (2, 4, 5 and 10 wt%). First, the appropriate amount of CeO_2_@HNT-NH_2_ was sonicated in 2-propanol at 10 W for 5 min. Then, 2.2 g of ionomer dispersion in water were stirred together with 0.5 g of DMAc for 1 h. Finally, CeO_2_@HNT-NH_2_ suspension in 2-propanol and Aquivion^®^ in DMAc were mixed and ultra-sonicated at 10 W for 5 min. After 10 min, the resulting suspension was cast on a Teflon sheet with a 200 µm blade. The membrane was treated in an oven at 80 °C for 16 hs in order to remove the solvent and afterwards annealed at 170 °C for 2 hs leading to a composite membrane 15 µm thick (measured with an electronic micrometer with resolution = ±0.001 mm and validated by scanning electron microscopy (SEM, (Hitachi Europe SAS, Velizy, France). For comparison purpose a 15 µm thick reference membrane of Aquivion^®^ 830 EW (10 wt%) was prepared by casting a solution prepared by mixing and stirring for 1 h a solution of 2.2 g of Aquivion^®^ 830 EW with 0.5 g DMAc and 2.3 g of 2-propanol on a Teflon sheet using a 200 µm blade. The PFSA membrane was submitted to the same thermal treatments used for the composite membrane. 

#### 2.3.1. Fenton Reaction Protocol

The chemical degradation of the membrane was accelerated by the Fenton reaction. 160 mg of the prepared membranes were immersed in a solution containing 45 mL of hydrogen peroxide (30% volume), 55 mL of ultrapure water, 7.5 µL of concentrated sulfuric acid and 28 mg of (NH_4_)_2_Fe(SO_4_)_2_(H_2_O)_6_. Then, the membrane was left in the Fenton reagent for 4 hs at 75 °C under reflux and with stirring. Afterwards, the solution was recovered and the TISAB III was added. A selective electrode was used to quantify the amount of fluoride ions released in the medium after the Fenton reaction.

#### 2.3.2. Membrane Characterisation

In-plane proton conductivity of the membranes was determined from resistance measurements made with a BT-512 BekkTech Conductivity Test System including a Keithley 2400 Sourcemeter (TeKtronix, Beaverton, OR, USA). The membrane samples (0.5 cm × 4 cm) were treated in sulfuric acid (1 mol/L) for 1 h and washed 3 times in deionised water for 15 min and left to dry overnight before being placed in the conductivity cell in contact with 4 platinum microelectrodes placed at a distance of 3.5 mm from each other. The temperature and relative humidity were controlled using the BT-201 Temperature Control System. The data were collected and analysed using BekkTech Conductivity Testing (BT512, FuelCellStore, Texas, USA). & LabView Data Analysis Software (8.1, National Instruments, Austin, TX, USA).

The concentration of fluoride ions released in the solution upon Fenton test (fluoride emission rate, FER) was obtained using an ion selective electrode (ISE, Thermo Scientific Orion Star Series Meter - ISO 10359-2: 1994, ThermoFisher Scientific, Waltham, USA). Prior to measurements, the electrode was calibrated using standard solutions at concentrations of 190 ppm, 1900 ppm and 19,000 ppm. The calibration line is validated when the slope is between −54 and −60 mV. The detection limit for this technique is *ca* 100 ppb.

The mechanical properties of the membranes were determined at 22 °C and a relative humidity of 40%. The tensile stress tests were carried out with a Z1.0 testing machine from Zwick Roell (Ulm, Germany), with a 200 N static sensor using at least three repetitions. The membranes were cut into 5 mm × 60 mm rectangles. Data were treated by the TestXpert Master software (11.0, Zwick Roell, Ulm, Germany).

## 3. Results and Discussion

### 3.1. Characterisation of HNTs

**Figure 1:**
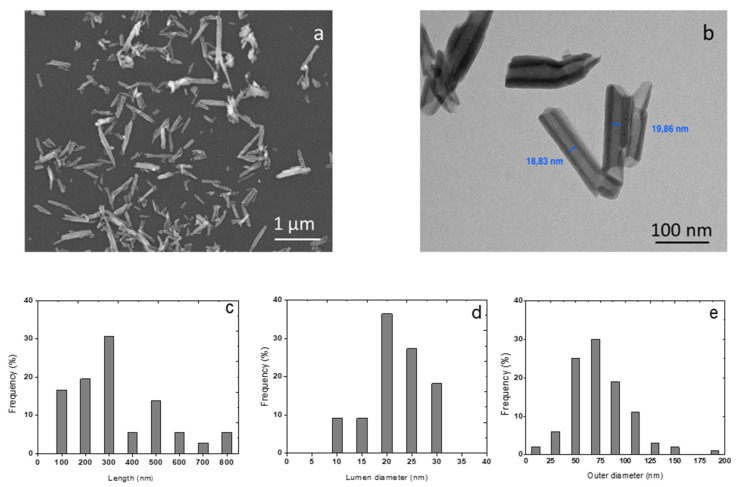
Scanning electron microscopy (SEM) (**a**) and transmission electron microscopy (TEM) micrographs of halloysite nanotubes (HNTs) (**b**) corresponding distribution histograms of length (**c**) *lumen* internal diameter (**d**) and outer diameter (**e**).

The morphology of halloysite clays was characterised by scanning and transmission electron microscopies (Figure 1). They presented a tubular structure with length varying with a wide dispersion from 100 nm to 800 nm. The average outer diameter measured 70 nm, while the average inner diameter (*lumen*) was 20 nm.

The structural analysis of the halloysite nanotubes (HNTs) (Figure S1a, Supplementary Materials) demonstrated peaks typical of halloysite [Al_2_Si_2_O_5_(OH)_4_] together with SiO_2_ and Fe_3_O_4_ co-crystallised into the kaolinite layers. A sharp peak at 12.1° corresponds to a basal spacing of 0.73 nm confirming the identity of the composite as dehydrated HNTs [58,59]. Other peaks with lower intensity at 20°, 24.5°, 35°, 37.9°, 54.5° and 62.5° also assigned to HNTs according to the standard JCPDS card no 00-029-1487 [60,61].

The chemical composition of HNTs was evaluated by XRF (Table 1) Together with the expected presence of Al and Si in agreement with XRD results, a non-negligible amount of iron was detected (0.34 wt%).

Iron is naturally present in halloysites. There is a correlation between the amount of iron present in halloysites and their morphology (flat, spherical, tubular) [62,63]. When the isomorphic substitution of Al^3+^ by Fe^3+^ increases, the curvature of the halloysite sheet decreases. Flat halloysites contain the largest amounts of Fe (from 2 to 6 wt%), while tubular halloysites relatively small amounts (from 0 to 3 wt%). Tubular halloysites are the most influenced by iron content with an inverse relationship between tube length and Fe content [55].

The presence of Fe^x+^ in HNTs is critical, as it may catalyse the formation of radicals and thus promote the chemical degradation of the clay-filled membranes. Treatment with oxalic acid, chosen for its acidic, reducing and chelating properties, [64,65] was performed in order to remove or reduce the amount of iron in the HNTs. XRF elemental analysis demonstrated that this treatment led to the reduction of iron content from 0.34 wt% to 0.23 wt% (Table 1) in HNTs without affecting their tubular morphology (Figure S2, Supplementary Materials). The crystal structure of halloysite was also unaffected by oxalic acid treatment. The diffractogram recorded after this treatment (Figure S1b, Supplementary Materials) is identical to that of untreated halloysite. This lack of structural change can be attributed to the use of a relatively low acid concentration for leaching compared to those reported by Panda et al. [63] and Zhang et al. [64]. The peak corresponding to the presence of FeO_x_ is still present in the diffractogram of the acid-treated HNTs and it is concluded that the remaining iron is structural, that is, occupies the Al^3+^ site in the clay structure [55]. Its possible activity as a catalyst for the Fenton reaction in the composite membrane will be described in paragraph 3.4. All the HNTs characterised and modified in this work were pre-treated with oxalic acid and contain the minimum amount of iron.

### 3.2. Preparation and Characterisation of CeO_2_@HNT 

In order to increase the chemical stability of the composite membrane embedding HNTs against attack of the hydroxyl HO• and hydroperoxy HOO• free radicals, which may be formed in the presence of multivalent cations leached from the fuel cell or iron from the same HNTs (Section 3.2), the clays were functionalised with cerium oxide nanoparticles.

HNTs have been modified with a range of active agents for their storage and release, especially for biomedical and pharmaceutical applications, either by intercalation between the sheets, adsorption on the external walls or encapsulation in the *lumen* [66,67,68]. The latter approach offers the highest capacity as well as the possibility of controlled release of the encapsulated entities. The empty HNT *lumen* corresponds to approximately 20 % of the total tube volume, which makes it suitable for loading with approximately 10 to 15 vol% of the active agents. In this work the embedding of CeO_2_ nanoparticles in the HNT *lumen* was adapted from a procedure developed by Abdullayev et al. [67] for insertion of the corrosion inhibitor benzotriazole. Firstly, ionic cerium in the form of nitrate was inserted in the inner porosity of HNT forming Ce@HNTs. From XRF analysis, the amount of cerium incorporated as salt was 8 wt%. A previous investigation on the kinetics of the release of the inorganic salt from the halloysite nanotubes evidenced the completion of the process in less than 24 hs (data not shown). This result can be explained by the increased mobility and solubility of inorganic ions in the release medium and their weak interaction with the HNT walls [69]. In order to stabilize cerium in the *lumen* and allow its slower and controlled release for a prolonged protection of the membrane from radical attack, Ce@HNTs was converted into cerium oxide nanoparticles (CeO_2_@HNTs) by thermal treatment in air [57]. It is known that CeO_2_ nanoparticles are formed by thermal decomposition of the hydrated nitrate precursor according to the following mechanism:

$$\mathrm{Ce}{\left({\mathrm{NO}}_{3}\right)}_{3}\;.\;6{\;H}_{2}O\;\overset{70–150\;{}^{\circ}C}{\to }\;\;\mathrm{Ce}{\left({\mathrm{NO}}_{3}\right)}_{3}\;.{\;H}_{2}O+5{\;H}_{2}O\;\mathrm{Ce}{\left({\mathrm{NO}}_{3}\right)}_{3}\;.{\;H}_{2}O\;\overset{150–225\;{}^{\circ}C}{\to }\;\mathrm{Ce}{\left({\mathrm{NO}}_{3}\right)}_{3}\;+{\;H}_{2}O\;\mathrm{Ce}{\left({\mathrm{NO}}_{3}\right)}_{3}\overset{225–400\;{}^{\circ}C}{\to }\;{\mathrm{CeO}}_{2}\;+{\;\mathrm{NO}}_{X}$$

At 300 °C the encapsulated cerium nitrate is considered fully decomposed to the corresponding oxide. The cerium loading was determined by XRF as 8 wt% (Table 1). XRD analysis (Supplementary Materials, Figure S1d) demonstrated the presence of CeO_2_ by the appearance of the corresponding peaks at 28.5°, 33°, 47.4° and 56.3° associated with reflections from (111), (200), (220), (311) planes of the fluorite cubic structure according to the JCPDS 01-075-0390 [31]. From the deconvolution of the high intensity peak at 28.5° (Supplementary Materials, Figure S3) and application of the Scherrer equation the size of the ceria nanoparticles was estimated to be 4–5 nm. In reasonable agreement, TEM analysis confirmed the presence of 3.5 nm cerium oxide particles located in the *lumen* (Supplementary Materials, Figure S4).

### 3.3. Preparation and Characterisation of CeO_2_@HNT-NH_2_

In order to improve the interface between the PFSA ionomer and the clay and thus the homogeneity of the clay dispersion in the membrane, the surface of the HNT was chemically modified by grafting of with an aminosilane agent (APTMS). Performed in anhydrous medium [70], this reaction gives rise to NH_2_ surface groups able to interact with the acidic functionalities of Aquivion^®^. The amount of APTMS grafted was quantified by comparing TGA traces of functionalised and bare HNT (Figure S5, Supplementary Materials). The two materials showed the same profile of degradation until 200 °C corresponding to the dehydration of the clays and a different mass loss at higher temperature attributed to the degradation of APTMS until 500 °C. The yield at 1200 °C of HNTs was 84.74% which is assigned to the inorganic components of HNTs, while for APTMS-HNTs was 79.08%. From the mass change values it is possible to estimate the amount of the APTMS grafted on the surface as 5.6 ± 0.2 wt%. The amount of nitrogen (from grafted APTMS) was also estimated by elemental analysis as being 1.3 wt%. 

**Figure 2:**
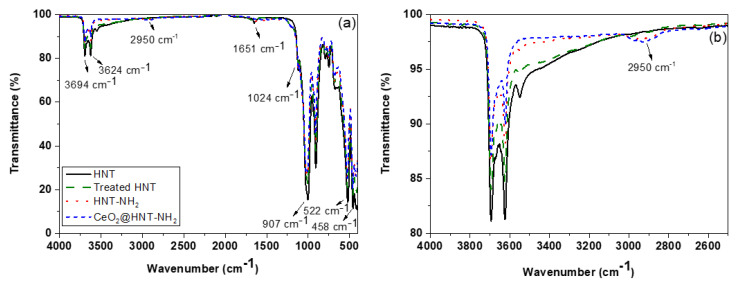
Fourier-transform infrared (FT-IR) spectra of HNTs, acid-treated HNTs, HNT_-_NH_2_-and CeO_2_@HNT-NH_2_ (**a**). In (**b**) is represented the enlarged 4000–2500 cm^−1^ region.

The Fourier-transform infrared (FT-IR) spectra of HNTs before and after functionalisation are shown in Figure 2. The absorption peaks at 3624 and 3694 cm^−1^ correspond to the OH stretching of inner surface hydroxyl groups and outer surface hydroxyl groups [56]. Other signals characteristic of HNT are displayed, such as deformation vibrations of Si–O–Si and Al–O–Si at 458 and 522 cm^−1^ and the inner Si–O stretching vibration at 1024 cm^−1^. The intensity of Al-O–H deformation vibration of the inner hydroxyl groups appears at 907 cm^−1^. The weak band at 1651 cm^−1^ is ascribed to the O–H deformation vibration of the adsorbed water.

After the functionalisation of HNTs with APTMS, the peak at 2950 cm^−1^ is assigned to the symmetric stretching vibration of –CH_2_ and the decrease in the intensity of the hydroxyl groups at 3624 and 3694 cm^−1^ demonstrated the presence of the coupling agent and the external surface functionalisation of HNTs.

Zeta potential measurements were performed at pH 7 after dispersing the HNTs in ultrapure water. Bare HNTs displayed a zeta potential of −26 mV, which corresponds to the charge of the silanol functionalities on the surface. SiO_2_ is negatively charged above pH 4. The zeta potential of HNT-NH_2_ was 27.2 mV at pH 7. The change of the charge value from a negative to a positive value after reaction with APTMS is in agreement with the effective grafting of NH_2_ moieties on the HNT external surface.

**Figure 3:**
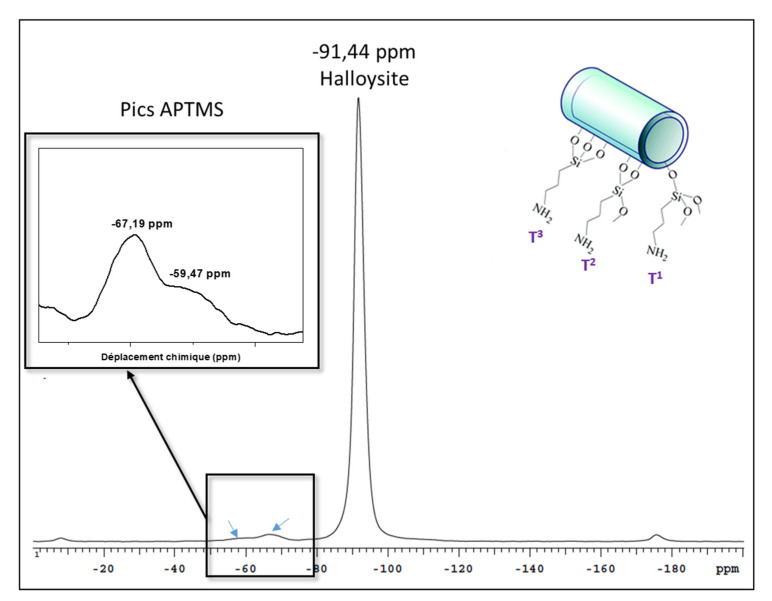
^29^Si NMR spectrum of HNT-NH_2._

In order to gain information on the nature of the coordination at the silicon atoms of the HNT surface, CP/MAS ^29^Si NMR analysis was carried out on HNT-NH_2_ and the spectrum obtained is presented in Figure 3.

It displays a high intensity peak at −92 ppm attributed to the presence of silicon Q^3^, Si(OSi)_3_(OAl) of HNTs. Low intensity signals at −68 ppm and −61 ppm correspond to the tridentate (T^3^) and bidentate (T^2^) coordination of silicon of the APTMS, respectively. Observation of the bidentate form of Si means that some APTMS species possess one methoxy or hydroxyl group that is not condensed [71]. 

Transmission electron microscopy observation was performed before and after the functionalisation of HNTs by cerium oxide particles and APTMS (Supplementary Materials, Figure S6). From TEM micrographs, the presence of CeO_2_ spherical particles inside the *lumen* with diameter ranging from 2 to 5 nm was demonstrated. Their average diameter of 3.5 nm is in agreement with the cerium oxide domain size determined from XRD (Supplementary Materials, Figures S1d and S3). The cerium oxide content of CeO_2_@HNT-NH_2_ after the reaction with APTMS **(**Table 1) was unchanged (8 wt%), demonstrating the stability of CeO_2_ nanoparticles in the *lumen*.

The results discussed so far demonstrate the effective formation of cerium oxide nanoparticles in the HNT *lumen* and the grafting of APTMS on the outer surface hydroxyl groups leading to the formation of a bi-functional material CeO_2_@HNT-NH_2_. The next section will present their incorporation into Aquivion^®^ ionomer to prepare nanocomposite membranes.

### 3.4. Composite Membrane Characterisation 

To investigate the effect of the residual iron in the halloysite nanoclays in the Fenton reaction, membranes of Aquivion^®^ 830 EW containing 20 wt% of as-received and acid-treated HNTs were prepared and compared with a reference membrane of Aquivion^®^ 830 EW. A greater amount of HNTs than that used in this work (20 vs. 10 wt%) was incorporated in the membranes to maximise the iron amount and therefore the release of fluoride ions from the ionomer according to the detection limit of the fluoride electrode.

**Figure 4:**
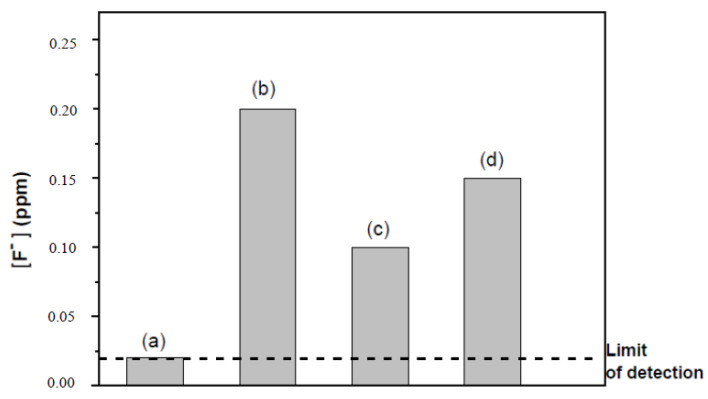
Histogram of the fluoride concentration released after 4 h in the Fenton reaction for the Aquivion^®^ membrane (without added Fe) (a) and composite membranes comprising untreated HNT (0.34 wt% Fe) (b) and acid-treated HNTs (0.23 wt% Fe) (c) and Aquivion^®^ membrane (with 0.23% of Fe added as (NH_4_)_2_Fe(SO_4_)_2_(H_2_O)_6_) (d).

When investigating the composite membranes no (NH_4_)_2_Fe(SO_4_)_2_(H_2_O)_6_ was added to the hydrogen peroxide solution (iron being already present in HNTs). During the study of the reference membrane in the Fenton reaction and for the purpose of comparison with the composite membrane containing the acid-treated HNTs, an amount of (NH_4_)_2_Fe(SO_4_)_2_(H_2_O)_6_ corresponding to 0.23 wt% of iron (as in the HNTs after oxalic acid treatment) was added. The reaction was followed by monitoring the amount of fluoride ion released (FER) in the medium after 4 hs (Figure 4). 

The reference Aquivion^®^ membrane immersed in a Fenton solution without Fe, showed no chemical degradation since the concentration of fluoride ions measured in the medium after 4 hs was 0.02 ppm, that is, within the detection limits of the ion-selective electrode (ISE). The composite membrane with pristine HNTs (containing 0.34 wt% of iron) released twice the amount of fluoride ions double (0.2 ppm) than the membrane containing the acid-treated HNTs (containing 0.23 wt% of iron) (0.1 ppm), while the ratio of the amounts of iron in the halloysites is less than 2 (1.48). On comparing the FER for the membrane containing the acid-treated HNTs and an Aquivion^®^ membrane in the presence of the same amount of iron (0.23 wt% by (NH_4_)_2_Fe(SO_4_)_2_(H_2_O)_6_ addition), it is concluded that the degradation induced by the residual iron in the HNTs is negligible (higher fluoride release for the Aquivion^®^ membrane in the presence of Fe^2+^). Therefore, structural iron has an effect in the production of radicals in the Fenton reaction but to a lower extent than free iron ions at the same concentration. The acidic treatment procedure was effective and the influence of the residual iron was negligible. From these results, the amount of clay incorporated in the membranes was reduced to 4 wt%, with radical scavenger incorporated into the HNTs, to counter the oxidative effect of structural iron. 

**Figure 5:**
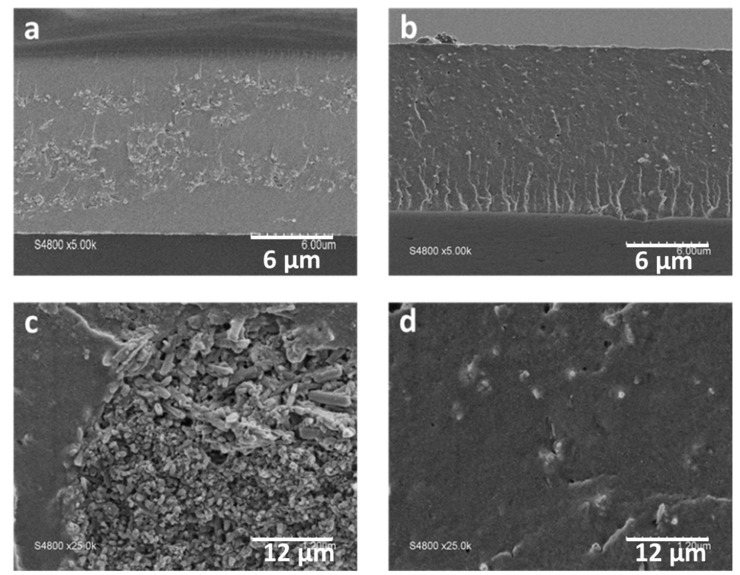
SEM images of the cross section of composite membrane with Aquivion^®^ loaded with 10 wt% HNT (**a**,**c**) and HNT-NH_2_ (**b**,**d**).

The dispersion and aggregation behaviour of halloysite are known to be highly related to the surface charge [72]. To verify this assumption, morphological analysis and proton conductivity determination were performed on the composite membranes embedding functionalised and non-functionalised nanoclays. From the SEM images of the cross section (Figure 5) of the composite membranes comprising 10% acid-treated HNT and CeO_2_@HNT-NH_2_ it may be seen that the latter presented higher compatibility with the ionomer. 

**Figure 6:**
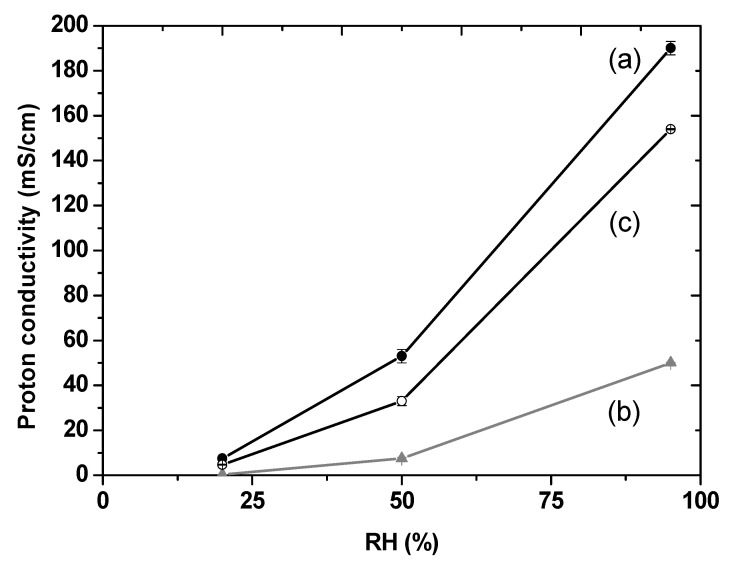
Proton conductivity measurements at 90 °C at different RH of pristine Aquivion^®^ membrane (a), composite membrane with 10 wt% loading of HNT (b) and 10 wt% loading of HNT-NH_2_ (c).

The homogeneity of the dispersion of the functionalised HNTs is observed throughout the thickness of the membrane (Figure 5b,d), while for the composite membrane comprising the same amount of HNTs, the presence of agglomerates of clays of micrometric size (2−3 µm) (Figure 5a,c) indicates poor interaction with the ionomer. The proton conductivity, Figure 6, of the reference Aquivion^®^ membrane at 90 °C and 95%RH (190 mS/cm) is in agreement with results reported elsewhere [73]. As a direct result of the poor dispersion, the incorporation of (non-functionalised) HNTs in Aquivion^®^ led to a significant decrease in the proton conductivity to 50 mS/cm, under the same conditions. Surface functionalisation of the HNT had a positive effect on the proton transfer in the composite membrane however, since the proton conductivity reaches 154 mS/cm at 90 °C and 95 %RH, only slightly lower than that the reference membrane, due to immobilisation of some of the protons in ionic crosslinking. The acid/basic interaction between the sulfonic groups of Aquivion^®^ and the amine groups of HNT-NH_2_ could lead to a decrease in the effective ion exchange capacity of the ionomer with a subsequent decrease in the conductivity of the corresponding membrane. 

The effect of the incorporation of CeO_2_@HNT-NH_2_ on the proton conduction and chemical stability properties of the corresponding membranes was investigated by preparing composite membranes with different amounts of CeO_2_@HNT-NH_2_ (the cerium loaded in the *lumen* being constant at 8 wt%). Composite membranes were prepared by casting a solution of Aquivion^®^ 830 EW (10 wt%) and different amounts of CeO_2_@HNT-NH_2_ from 2 to 10 wt% corresponding to Ce/HSO_3_^−^ mole ratio from 1% to 5 %. The molar ratio of cerium to HSO_3_^−^ groups in Aquivion^®^ PFSA ratio is a straightforward method to compare immobilised radical scavenger. It has been reported that 1% led to a significant (7 times) increase in durability in fuel cell measurement [47]. 

**Figure 7:**
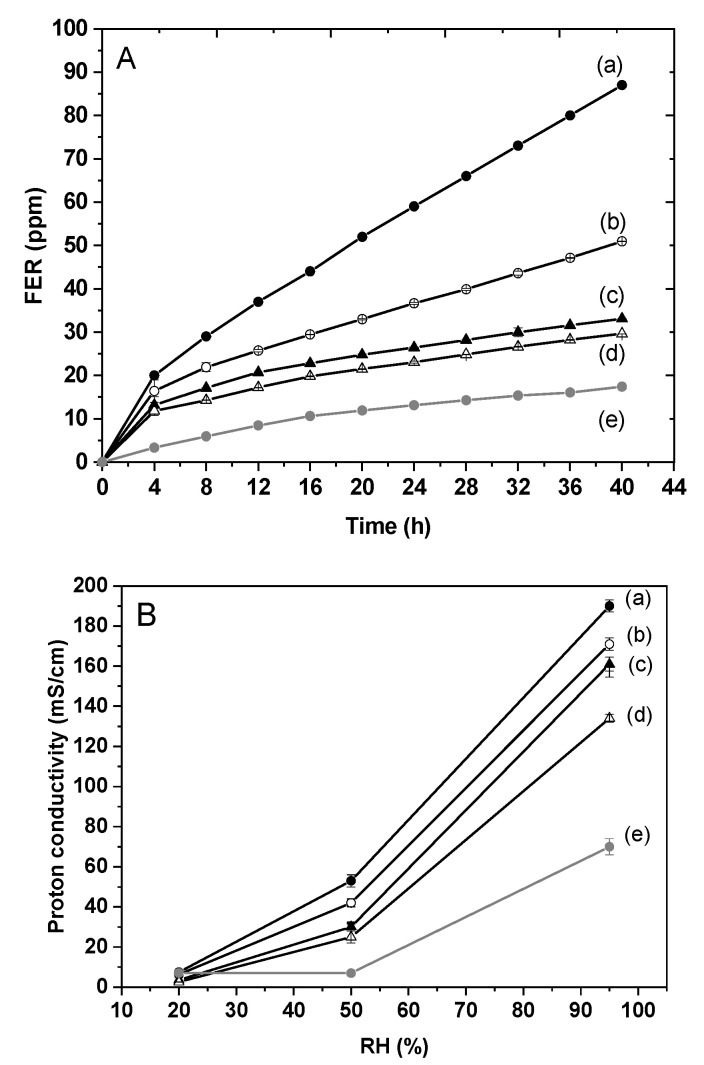
Fluoride Emission Rate (FER) as a function of time (**A**) and in-plane proton conductivity at 90 °C as a function relative humidity (**B**) of composite membranes loaded with different amounts of CeO_2_@HNT-NH_2_ corresponding to the following Ce/HSO_3_^−^ ratios: 1% (b), 2% (c), 2.5% (d), 5% (e) and comparison with the pristine Aquivion^®^ membrane (a).

The chemical stability of the above composite membranes was evaluated in a Fenton test and the quantity of fluoride ions released over time (FER) was measured after every 4 hs. The Fenton solution was renewed each time in order to ensure the same conditions throughout the experiment. The time-dependent FER for all composite membranes is depicted in Figure 7. The higher the quantity of cerium incorporated into the membranes, the lower the concentration of F^−^ measured in the Fenton solution and therefore the higher the chemical stability of the membrane against radical attack. However, higher CeO_2_@HNT-NH_2_ content in the membrane leads to lower proton conductivity, which could be explained by a release of cerium ions from the nanometric oxide encapsulated in halloysite and its migration in the membrane, blocking proton transfer sites [44,45,46,74,75].

For further study, membranes containing 4 wt% of CeO_2_@HNT-NH_2_, corresponding to 2 mol% Ce/HSO_3_^−^ ratio, was selected since it enabled high proton conductivity (160 mS/cm) and significant scavenging activity. This Ce/HSO_3_^−^ ratio corresponds to that considered as optimum in a study on silica-immobilised ceria radical scavenger [47]. The microscopy images of the bi-functional clays and of the cross-section of the corresponding composite membranes are depicted in Figure S6 of Supplementary Materials. The dispersion of the nanomaterials is homogeneous all over the membrane thickness. 

Tensile stress/strain measurements were performed on the Aquivion^®^ 830 EW reference membrane and the composite membranes containing 4 and 10 wt% of CeO_2_@HNT-NH_2_. The stress/strain curves are shown in Figure S7 of the Supplementary Materials and the corresponding calculated mechanical properties (Young modulus, yield stress, breaking strength) are summarised in Table 2. Average mechanical properties derived from stress/strain test conducted at 22 °C and 40 % RH on reference and composite membranes.

All membranes have ductile mechanical behaviour. The reference membrane has a higher elongation at break (160%) followed by the membrane loaded with 4 wt% (93%) and 10 wt% (77%) of CeO_2_@HNT-NH_2_. This result is expected because the incorporation of an inorganic component in a polymer matrix generally leads to increased hardness of the resulting composite material [42,43]. The ductility of the membrane therefore decreases with the rate of incorporated functionalised HNTs. Since the dimensional change of membranes in the plane of the membrane during fuel cell operation is always significantly lower than these stress at break values, more pertinent indicators are the Young’s modulus and the yield stress, which both increased significantly (+85% and 26%, respectively) for the composite membrane with 10 wt% CeO_2_@HNT-NH_2_. The selected composite membrane loaded with 4 wt% of functionalised clays presented very similar values of mechanical property indicators to those of the reference membrane. 

It is concluded that the incorporation of 4 wt% CeO_2_@HNT-NH_2_ in Aquivion^®^ did not lead to significant effect on the membrane mechanical properties. The composite membrane with such composition presented high proton conductivity and significant increase in chemical stability against free radicals, demonstrating the effectiveness of the approach. In situ investigation in a single fuel cell will further validate the strategy of immobilisation and release of radical scavengers in PFSA membranes for their enhanced lifetime. 

## 4. Conclusions

With the aim of enhancing their chemical and mechanical stability, composite proton-exchange membranes incorporating radical scavengers immobilized in nanoclays were prepared and characterized. Bi-functional halloysites, grafted with amino groups and embedding CeO_2_ nanoparticles (CeO_2_@HNT-NH_2_), were used as nanocontainers to immobilise and release the radical scavenger to the Aquivion^®^ ionomer. Composite membranes incorporating 4 wt% CeO_2_@HNT-NH_2_ presented unchanged tensile properties but high proton conductivity and increased stability to radical attack compared to non-modified Aquivion^®^ membranes, demonstrating the effectiveness of the approach. In situ characterisation in a single fuel cell will further validate it and specific approaches to improve mechanical resistance are currently under investigation.

## Figures and Tables

**Table 1:** Chemical composition of the pristine HNTs, acid-treated HNTs, CeO_2_@HNTs and CeO_2_@HNT-NH_2_ materials determined by X-Ray Fluorescence (XRF) and elemental analysis.

Material	Al (wt %)	Si (wt %)	O (wt %)	N (wt %)	Fe (wt %)	Ce (wt %)
HNT	21.85	22.00	55.15	-	0.34	-
treated HNT	18.39	21.36	56.57	-	0.23	-
CeO_2_@HNT	18.39	21.36	56.57	-	0.23	8.0
CeO_2_@HNT-NH_2_	11.21	16.21	72.40	1.30	0.23	8.0

**Table 2:** Mechanical properties of reference membrane Aquivion^®^ 830EW and composite membrane Aquivion^®^ + 4 % CeO_2_@HNT-NH_2_ and Aquivion^®^ + 10 % CeO_2_@HNT-NH_2._

Membrane	Young Modulus (N/mm²)	Breaking Strength (N mm²)	Yield Stress (N mm²)
Aquivion^®^ 830 EW	238 ± 7	14 ± 0.7	10 ± 0.3
Aquivion^®^ + 4 % CeO_2_@HNT-NH_2_	237 ± 5	11 ± 1.3	9 ± 0.6
Aquivion^®^ + 10 % CeO_2_@HNT-NH_2_	326 ± 9	15 ± 1.5	12 ± 0.3

## Supplementary Materials

The following are available online at https://www.mdpi.com/2077-0375/10/9/208/s1, Figure S1: X-ray diffraction of pristine HNT, acid-treated HNT, HNT-NH_2_ and CeO_2_@HNT materials; Figure S2: SEM micrograph of acid-treated HNTs; Figure S3: Deconvolution of the high intensity peak of CeO_2_ in the X-ray diffractogram of CeO_2_@HNT; Figure S4. TEM micrographs of HNTs (a), CeO_2_@HNT-NH_2_ (b) and histogram of diameter size of cerium oxide particles (c); Figure S5. TGA curves of acid-treated HNTs and HNTs-NH_2_; Figure S6. SEM micrographs of the bi-functional CeO_2_@HNT-NH_2_ clays (a) and of the cross-section of the corresponding composite membrane loaded at 4 wt% (2 mol% Ce/HSO_3_^−^); Figure S7. Stress/strain test curves registered at 22 °C and 40% RH on reference and composite membranes.
