# Supplementary material for: Immobilisation and Release of Radical Scavengers on Nanoclays for Chemical Reinforcement of Proton Exchange Membranes
Source: Membranes (Basel). 2020 Aug 28;10(9):208. doi: 10.3390/membranes10090208 (PMC7559798; doi:10.3390/membranes10090208)
Supplement: Supplementary file 1 [file membranes-10-00208-s001.pdf]

Supplementary Materials

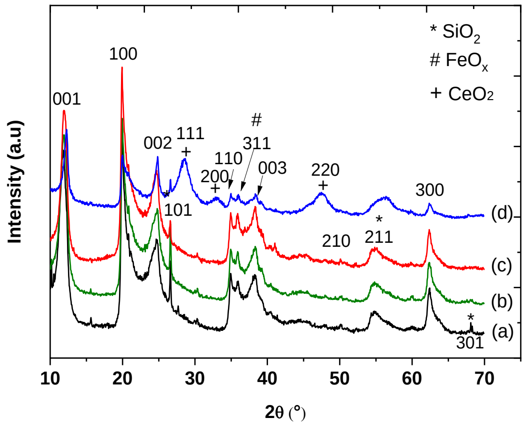

Figure S1. X-ray diffraction of pristine HNT (a), acid-treated HNT (b), HNT-NH_2 (c) and CeO_2@HNT (d) materials.

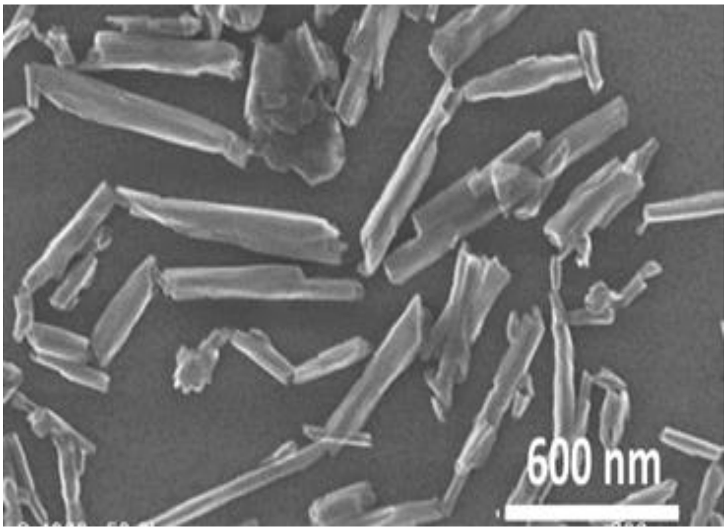

Figure S2. SEM micrograph of acid-treated HNTs.

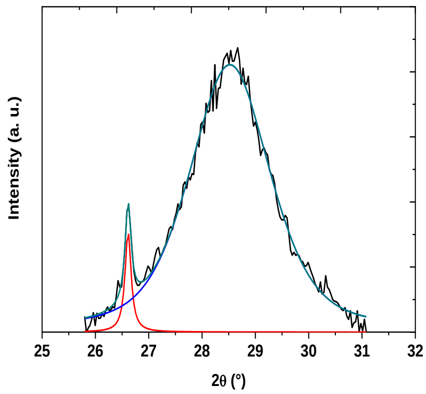

Figure S3. Deconvolution of the high intensity peak of CeO_2 in the X-ray diffractogram of CeO_2@HNT

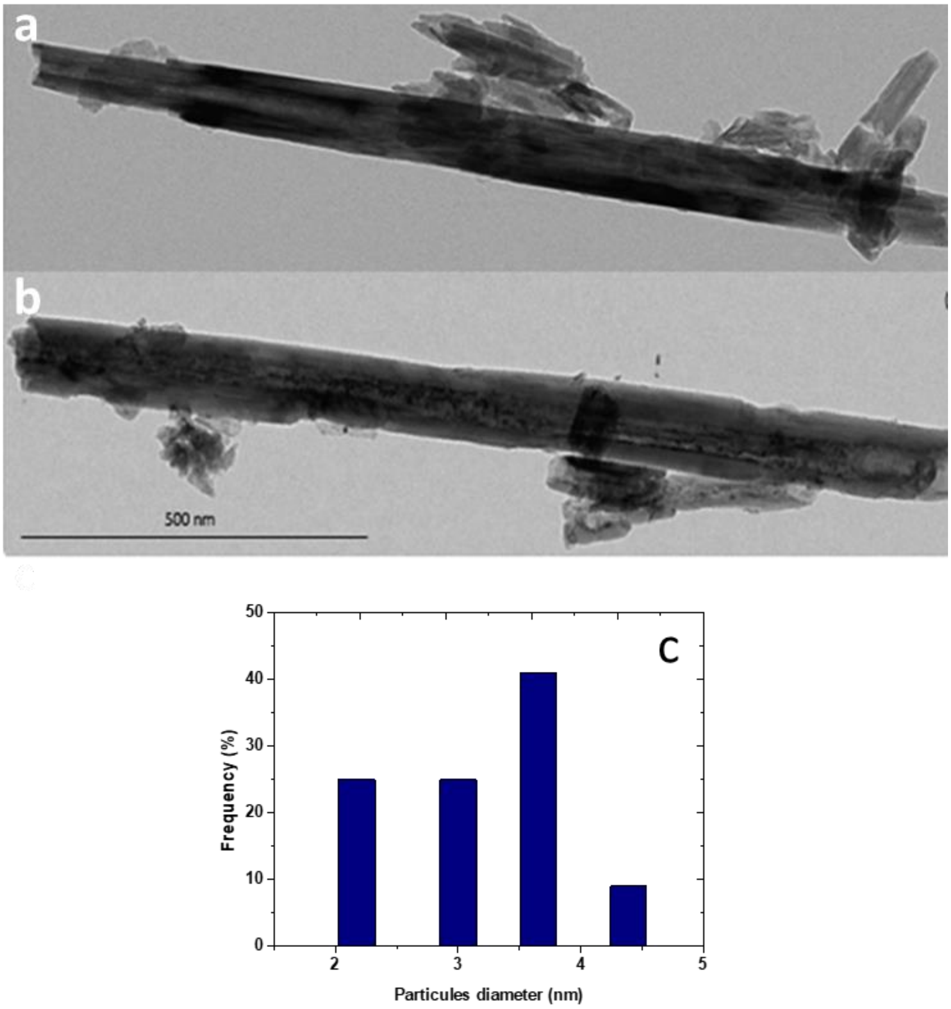

Figure S4. TEM micrographs of HNTs (a), CeO_2@HNT-NH_2 (b) and histogram of diameter size of cerium oxide particles (c).

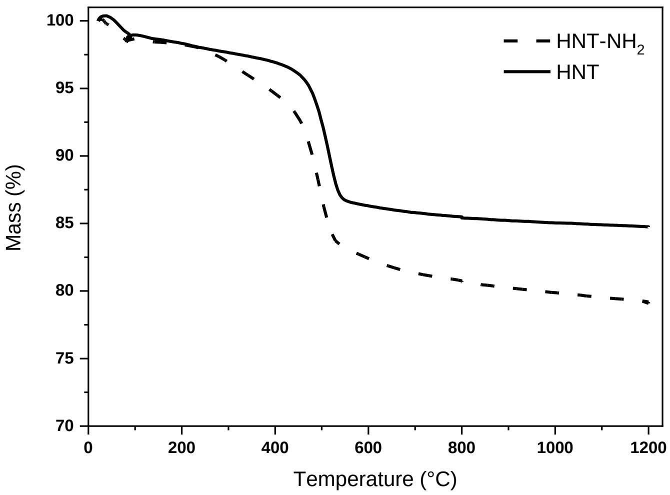

Figure S5. TGA curves of acid-treated HNTs and HNTs-NH_2.

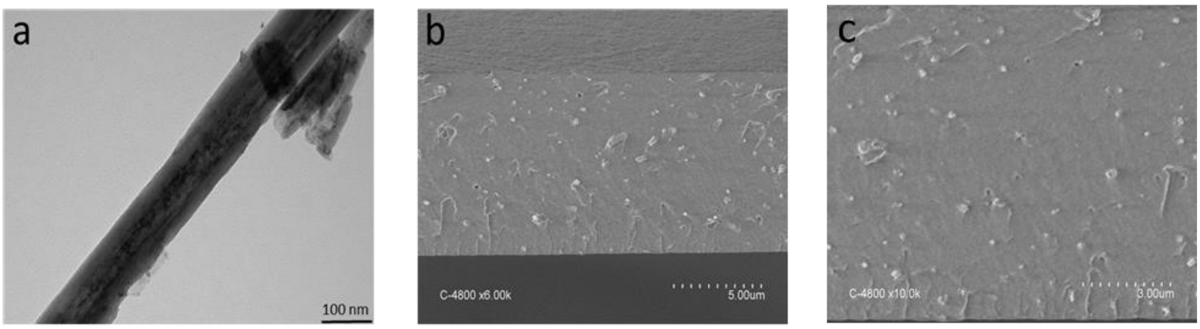

Figure S6. SEM micrographs of the bi-functional CeO_2@HNT-NH_2 clays (a) and of the cross-section of the corresponding composite membrane loaded at 4 wt% (2 mol% Ce/HSO_3^-).

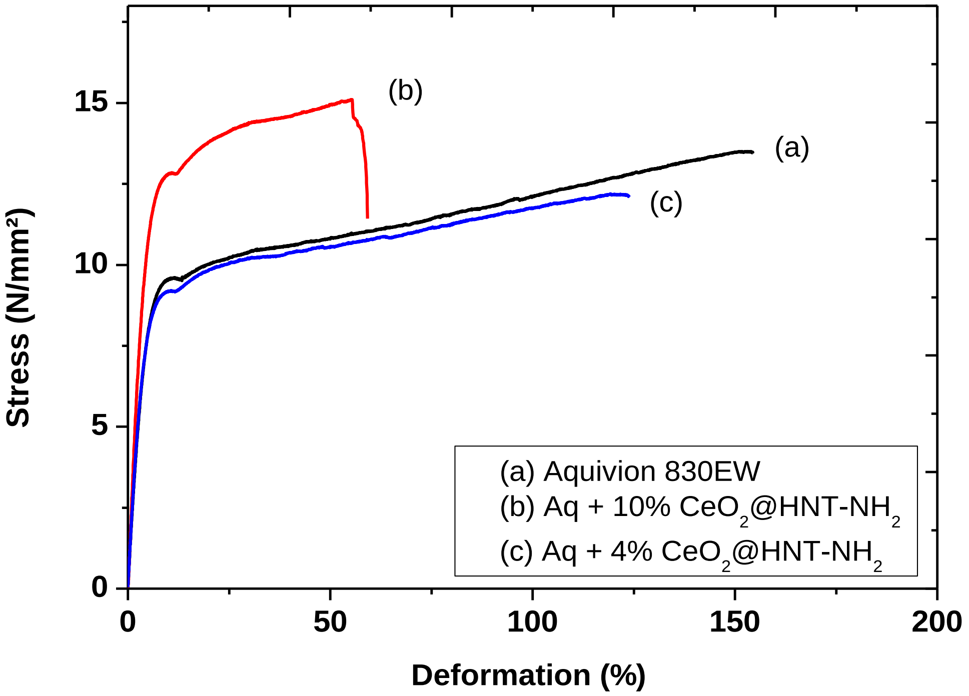

Figure S7. Stress/strain test curves registered at 22 °C and 40 % RH on reference and composite membranes.
